# Neuropathological stages of neuronal, astrocytic and oligodendrocytic alpha-synuclein pathology in Parkinson’s disease

**DOI:** 10.1186/s40478-025-01944-x

**Published:** 2025-02-11

**Authors:** Maria Otero-Jimenez, Marcelina J. Wojewska, Lawrence P. Binding, Simona Jogaudaite, Sandra Gray-Rodriguez, Alexandra L. Young, Steve Gentleman, Javier Alegre-Abarrategui

**Affiliations:** 1 Department of Brain Sciences, Imperial College London, Hammersmith Hospital, London, UK; 2 Department of Computer Science, UCL Hawkes Institute, University College London, London, UK

**Keywords:** Alpha-synuclein, Alpha-synucleinopathies, Parkinson’s disease, Astrocytes, Neuroanatomical distribution, Staging

## Abstract

Alpha-synucleinopathies are neurodegenerative diseases characterized by the spread of alpha-synuclein (α-syn) aggregates throughout the central nervous system in a stereotypical manner. These diseases include Lewy body disease (LBD), which encompass Dementia with Lewy bodies (DLB), Parkinson’s Disease (PD), and Parkinson’s Disease Dementia (PDD), and Multiple System Atrophy (MSA). LBD and MSA chiefly contain α-syn aggregates in neurons and oligodendrocytes, respectively, although glial α-syn pathology in LBD is increasingly being recognized. Semi-quantitative and machine learning-based quantifications of neuronal, oligodendrocytic and astrocytic α-syn pathology were implemented on a cohort of LBD and MSA post-mortem tissue samples. The neuroanatomical distribution of each cell-type specific α-syn pathology was evaluated using conditional probability matrices and Subtype and Stage Inference (SuStaIn) algorithm. We revealed extensive glial α-syn pathology in LBD, emphasizing the disease- and region-specific profile of astrocytic α-syn pathology, which was absent in MSA and minimal in the substantia nigra of LBD. Furthermore, we have described distinct morphologies of astrocytic α-syn pathology, which were found to correlate with the density of astrocytic α-syn inclusions. Astrocytic α-syn pathology was mainly centered in the amygdala and exhibited a unique stereotypical progression whilst oligodendrocytes displayed a distribution akin to the established neuronal progression pattern. SuStaIn modeling was further used to test for heterogeneity in the spatiotemporal progression, revealing that a subset of cases might follow an alternative pattern. Based on these findings, we introduce a novel multimodal progression framework that integrates, for the first time, the temporal and spatial progression of astrocytic and oligodendrocytic α-syn pathology alongside neuronal pathology in PD, providing further information regarding the role of neurons and glia in disease pathogenesis.

**Supplementary Information:**

The online version contains supplementary material available at 10.1186/s40478-025-01944-x.

## Introduction

Alpha-synucleinopathies (α-synucleinopathies) are neurodegenerative diseases characterized by the accumulation of pathological alpha-synuclein (α-syn) aggregates and neuronal cell death [12]. α-Synucleinopathies include Dementia with Lewy Bodies (DLB), Parkinson’s Disease (PD), Parkinson’s Disease Dementia (PDD) and Multiple System Atrophy (MSA). The neuropathological hallmark in PD, PDD and DLB, collectively termed Lewy body disease (LBD), is the presence of α-syn aggregates in neurons named Lewy bodies (LBs) and Lewy neurites (LNs) [33]. MSA is characterized by the presence of α-syn aggregates in oligodendrocytes in the form of glial cytoplasmic inclusions (GCIs) [14, 47]. Neuronal α-syn inclusions, while less common than GCIs, are also observed in MSA and can manifest in brain regions typically spared by GCI pathology [14]. Analogously, glial α-syn pathology has been reported in LBD [3, 10, 19, 42] with relatively few reports of oligodendrocytic and astrocytic α-syn pathology [10]. Clinically, up to 80% of PD patients will eventually develop dementia [20] whilst DLB accounts for 10–15% of the total number of people living with dementia [32]. MSA is the most aggressive of all α-synucleinopathies, with a fast disease progression and an average age of onset around 50 years old [36].

Physiologically, α-Syn is a 14 kDa protein predominantly found in the presynaptic terminals [38]. Abnormal misfolded species are capable of forming pathological aggregates and spreading in a prion-like manner [37]. The fact that aggregates of α-syn spread throughout the brain in a stereotypical pattern led to the PD staging system, initially proposed by Braak in 2003, describing the neuroanatomical distribution of neuronal α-syn lesions throughout the brain [15]. In summary, according to this system, neuronal lesions first appear in the dorsal motor nucleus X (DMX) of the medulla oblongata and in the locus coeruleus of the pons in stages 1 and 2, respectively. By stage 3, α-syn pathology spreads to the substantia nigra (SN) in the midbrain, further involving the anteromedial temporal mesocortex (transentorhinal region and amygdala) by stage 4. Neocortical areas, such as higher-order and first-order sensory association areas are affected by stage 5 and 6, respectively [9]. Since the introduction of Braak staging, alternative staging systems have been proposed including the Unified Staging System by Beach [7] and the LP consensus criteria [4] allowing for the classification of atypical cases that do not follow the classical progression of α-syn pathology. Nevertheless, existing staging systems focus on the neuronal component of α-syn pathology leaving a significant gap in understanding the comprehensive spatiotemporal progression of α-syn across different cell populations and α-synucleinopathies.

Glial α-syn pathology in LBD has been previously underrecognized in human post-mortem brain tissue. Astrocytic α-syn pathology has been documented chiefly in the amygdala and other limbic areas in cases with LBD with a distribution largely described parallel to neuronal pathology which became more abundant in more advanced disease stages [10, 45, 50, 51]. The presence of astrocytic α-syn pathology in MSA [44] has been minimally studied, with only one paper reporting the accumulation of phosphorylated α-syn in subpial and subependymal astrocytes [34]. An antibody recognizing the non-amyloid beta component of AD amyloid (NAC) region and formic acid antigen retrieval are required to label astrocytic α-syn pathology in LBD [10, 43]. Additionally, astrocytic α-syn pathology has been shown to be negative for ubiquitin and p62, canonical markers of aggregated proteins [3], as well as for α-syn phosphorylated at serine 129 [44]. Recent findings indicate that astrocytic α-syn inclusions are likely truncated at both the N- and C-termini [3, 43] explaining the inability of C-terminal-specific antibodies to detect these inclusions [3, 46]. Moreover, their sensitivity to proteinase K digestion suggests that these aggregates might be composed of a combination of soluble and insoluble α-syn species [3].

In this study we aimed to investigate the neuroanatomical distribution of cell-type specific α-syn pathology in α-synucleinopathies, focusing on glial pathology. We demonstrate that glial α-syn pathology is widespread in LBD cases, with astrocytic α-syn pathology displaying unique features including its notable absence in MSA. Moreover, we characterized a spectrum of novel astrocytic α-syn morphologies which correlate with the severity of astrocytic α-syn pathology. Astrocytic α-syn pathology displays a distinct stereotypical pattern of progression in LBD differing from that observed in oligodendrocytes, which resembled the classic distribution of neuronal α-syn in established staging systems. Given the separate temporal and spatial progression patterns of oligodendrocytic and astrocytic α-syn pathology, we have proposed a novel multimodal staging model for PD/PDD incorporating these cell-type specific pathological events into the neuronal model.

## Materials and methods

### Post-mortem human tissue

The cohort was composed of MSA (*n* = 15), DLB (*n* = 14), prodromal PD Braak stages 1/2 (*n* = 2), early PD Braak stages 3/4 (*n* = 16), late PD Braak stages 5/6 (*n* = 20) and PDD (clinically reported; *n* = 18). Formalin-fixed paraffin-embedded (FFPE) post-mortem human tissue was obtained from The Multiple Sclerosis and Parkinson’s UK Tissue Bank at Imperial College London and used under Research Ethics Committee Approval Ref. No 07/MRE09/72. Regions analyzed included the amygdala, midbrain, medulla, cerebellum and superior frontal, anterior cingulate, parietal, and entorhinal cortices. Detailed cohort information can be found in (see Supplementary Table 1, Additional file 1).

### Immunohistochemistry

FFPE tissue was sectioned with a microtome at a thickness of 5 μm. FFPE-tissue sections were baked at 55ºC for 45 min before being dewaxed in xylene and rehydrated in descending concentrations of ethanol. The endogenous peroxidase activity was blocked in 0.3% H_2_O_2_ for 30 min at room temperature (RT) and antigen retrieval was performed in 100% formic acid for 10 min. The tissue sections were blocked in 10% normal horse serum (NHS; 2B Scientific, S-2000-20) and incubated with primary antibody overnight at 4ºC (1:1000 recognizing NAC α-syn region (clone 42); BD Biosciences, 610786). Next day, the slides were incubated with ImmPRESS[R] HRP Horse Anti-Mouse IgG Polymer Detection Kit (2B Scientific, MP-7402) for 30 min at RT. ImmPact DAB Substrate HRP (2B Scientific, SK-4105) was then added, and sections were counterstained with Haematoxylin (TCS Biosciences, HS315), dehydrated with ethanol and xylene, before being coverslipped using the Epredia™ ClearVue™ Coverslipper.

Images were acquired with a DS-Fi2 Digital Camera attached to a Nikon Eclipse 50i microscope using a DS‐U3 Digital Camera Controller and NIS‐Elements image acquisition software.

### Neuropathological analysis

Stained tissue sections for α-syn were scanned using Leica Aperio AT2 slide scanner. The whole slide images (WSIs) were assessed by 3 raters (MOJ, MJW and JAA) using QuPath software (version 0.4.4.) [6] for navigation across the virtual slide at x40 magnification comparable to a 40× microscope objective. Cases with oligodendrocytic and astrocytic α-syn pathology were classified using a semi-quantitative scale as mild (1 inclusions per 40x field of view), moderate (2–3 inclusions per 40x field of view) or severe (more than 3 inclusions per 40x field of view). The raters were blinded to any clinical or neuropathological diagnosis and used the proposed semi-quantitative scoring guidelines to classify the severity of oligodendrocytic and astrocytic α-syn inclusions.

### Immunofluorescence

Tissue sections were dewaxed and rehydrated as previously described. Antigen retrieval was performed (100% formic acid, heat-mediated in citrate buffer, pH 6.0) before blocking for 1 h at RT in 10% normal donkey serum (NDS; 2B Scientific, SP-072-VX10). Primary antibodies were incubated overnight at 4ºC 1:250 α-syn, (BD Biosciences, clone 42); 1:1000 GFAP (Invitrogen, 13–0300); 1:500 Aldh1l1 (abcam, ab190298). Next day, sections were incubated for 2 h at RT in a humidified chamber with 10% NDS and 1:1000 secondary antibodies (Donkey Anti-Mouse Alexa Fluor™ 488 (Invitrogen, A2120), Donkey Anti-Rabbit Alexa Fluor™ 647 (Invitrogen, A31573) and Donkey Anti-Rat Alexa Fluor™ 555 (Invitrogen, A21434)). Tissue sections were mounted with VECTASHIELD^®^ Antifade Mounting Medium with DAPI (Vector Laboratories, H-1200-10).

Fluorescence images were acquired using an Olympus BX63 scanning fluorescence microscope implementing the cellSens imaging software. FIJI (version 2.3.0.) was used for the processing of fluorescence images.

### Conditional probability matrix

All cases and brain regions were classified according to the presence or absence of α-syn pathology in oligodendrocytes, astrocytes and neurons (a single cell-type specific α-syn inclusion was considered as present). The conditional probability matrix was performed to investigate the probability of a given brain region having α-syn pathology in the absence of α-syn pathology in another brain region, as previously described [21, 24]. In brief, the percentage of cases with α-syn pathology being present (α-syn +) or absent (α-syn -) in two regions (X and Y) was calculated. Then, the conditional probability of region X being positive when Y is negative was calculated as and the probability of region Y being positive when X is negative was calculated as previously reported [21, 23, 24] (Table 1).

McNemar’s test was used to assess the evidence against the null hypothesis. The significance was set at *p* < 0.05 although results with significance of *p* < 0.09 are also shown. The same procedure was followed to analyze the conditional probability of a cell-type specific α-syn inclusion preceding another in each region.

$$\:P\left(X|Y\right)=\frac{b}{(b+d)}$$

$$\:P\left(Y|X\right)=\frac{c}{(c+d)}$$

**Table 1:** Formula used for calculating the conditional probability matrix

	Region Y	
Region X	α-syn +	α-syn -
α-syn +	*a*	*b*
α-syn -	*c*	*d*

The percentage of cases with α-syn pathology being present (α-syn +) or absent (α-syn -) in regions X and Y was calculated. Then, the conditional probability of region X being positive when Y is negative was calculated as b/(b + d), and the conditional probability of region Y being positive when Y is negative was calculated as c/(c + d).

### Machine learning algorithm for detection of cell-specific perikaryal α-syn inclusions

A machine learning based-approach was developed for the differential detection and quantification of cell-type specific perikaryal α-syn inclusions using QuPath software (version 0.4.4.) [6]. The categorization of cell-type specific perikaryal α-syn inclusions was performed by microscopic inspection of tissue sections (stained for α-syn). The categorization was based on parameters such as cell and nucleus size, and chromatin condensation. A visual template was created based on these parameters, allowing for accurate and replicable categorization (see Supplementary Fig. 1, Additional file 1). Stained tissue sections were scanned and used for the development and analysis with the machine learning algorithm. The machine learning-based classifiers were developed using artificial neural networks (ANN). For this, scanned tissue sections were used, and cells were first detected using StarDist (version 0.3.2.) deep learning approach [49]. Cells were manually annotated and classified into each cell type as positive or negative for α-syn pathology in minimum 10 whole slide images (WSIs) per brain region. The classifiers were validated in a separate dataset into initially 2 WSIs per brain region. All WSIs and the classification made by the machine learning algorithm were manually validated.

### SuStaIn analysis

The Subtype and Stage Inference algorithm (SuStaIn) [2, 52–54] was applied to the PD/PDD cohort to infer the temporal pattern of progression of α-syn in PD/PDD and test for heterogeneity in this temporal progression pattern across individuals. SuStaIn was applied separately to α-syn pathology ratings in neurons, oligodendrocytes and astrocytes to determine the progression of neuronal, oligodendrocytic, and astrocytic α-syn pathology. In each case, SuStaIn was first applied assuming a single common progression pattern across individuals to determine the overall progression of α-syn pathology. This assumption was then relaxed by applying SuStaIn assuming the existence of multiple patterns and thus tested whether there were individuals who followed an alternative progression pattern. The data used in Ordinal SuStaIn was scores from 1 to 5 representing the number of cellular α-syn inclusions (in the medulla (reticular formation), midbrain (SN), amygdala, cingulate, frontal and parietal cortices) obtained via our ANN. Cut offs of 1, 2, 4, 6 and 6 + neuronal and oligodendrocytic inclusions, and cut offs of 2, 4, 6, 8 and 8 + astrocytic inclusions, were used to assign scores of 0–5, respectively. Ordinal SuStaIn requires as input the probability each region has each score in an individual. Following methods previously described in [54], we evaluated these probabilities using a Gaussian distribution with a mean of the assigned regional score (0–5) and standard deviation of 0.5. This corresponds to the assumption that approximately 80% of the regional scores are representative of the true underlying score, 20% of the scores differ by 1, and 1% have a true score that has a difference greater than 1. The uncertainty in the ordering of biomarkers was evaluated by Markov Chain Monte Carlo (MCMC) sampling. The progression patterns and their uncertainty were visualized using positional variance diagrams with different colors representing a low to high severity of α-syn pathology: red (1), pink (2), blue (3), purple (4) and turquoise (5).

### Statistical analysis

GraphPad Prism software (version 10.2.2.) was used for statistical analysis. Data was shown as bars representing mean ± standard error of the mean (SEM). One-way or two-way ANOVA was performed followed by Tukey’s post-hoc test. Statistical significance was set at *p* < 0.05.

Fisher’s exact test was used for the correlation of astrocytic α-syn pathology and cognitive impairment, and Mann-Whitney test for correlating astrocytic α-syn pathology with disease duration and age of onset.

## Results

### Astrocytic α-syn pathology is most prevalent in the limbic system of LBD cases and it is negligible in MSA

α-Synucleinopathies differ in the types of cells primarily affected by α-syn pathology although both glial and neuronal α-syn pathology have been reported in PD/PDD, DLB and MSA cases. For a detailed investigation of cell-type specific α-syn pathology, perikaryal α-syn inclusions in oligodendrocytes, astrocytes and neurons were analyzed separately by discriminating based on parameters such as cell and nucleus size, and chromatin condensation. In brief, oligodendrocytes had a small, round nucleus with condensed chromatin and α-syn inclusions had a punctiform or GCI-like morphology (half-moon, sickle-shaped or flame-like α-syn inclusions), which were adjacent to or overlapped the nucleus, (matching the size of an oligodendrocyte nucleus or smaller) and were found next to neurons. Astrocytes had rounder or oval and larger nuclei with pale chromatin, and α-syn inclusions were observed as ramifications with a bushy-like morphology or as punctate densely packed staining. Neurons had oval shaped nuclei with unpacked chromatin, and α-syn inclusions appeared either adjacent to the nucleus, covering the cytoplasm, as rounded inclusions overlapping the nucleus or as LBs. Neuritic α-syn pathology such as LNs were not analyzed due to the absence of a trackable nucleus (see Supplementary Fig. 1, Additional file 1). Microglia were excluded in the analysis as α-syn inclusions were minimal and scarcely detected by IHC. The presence of astrocytic α-syn inclusions was confirmed using several astrocyte markers (see Supplementary Fig. 2, Additional file 1).

**Fig. 1:**
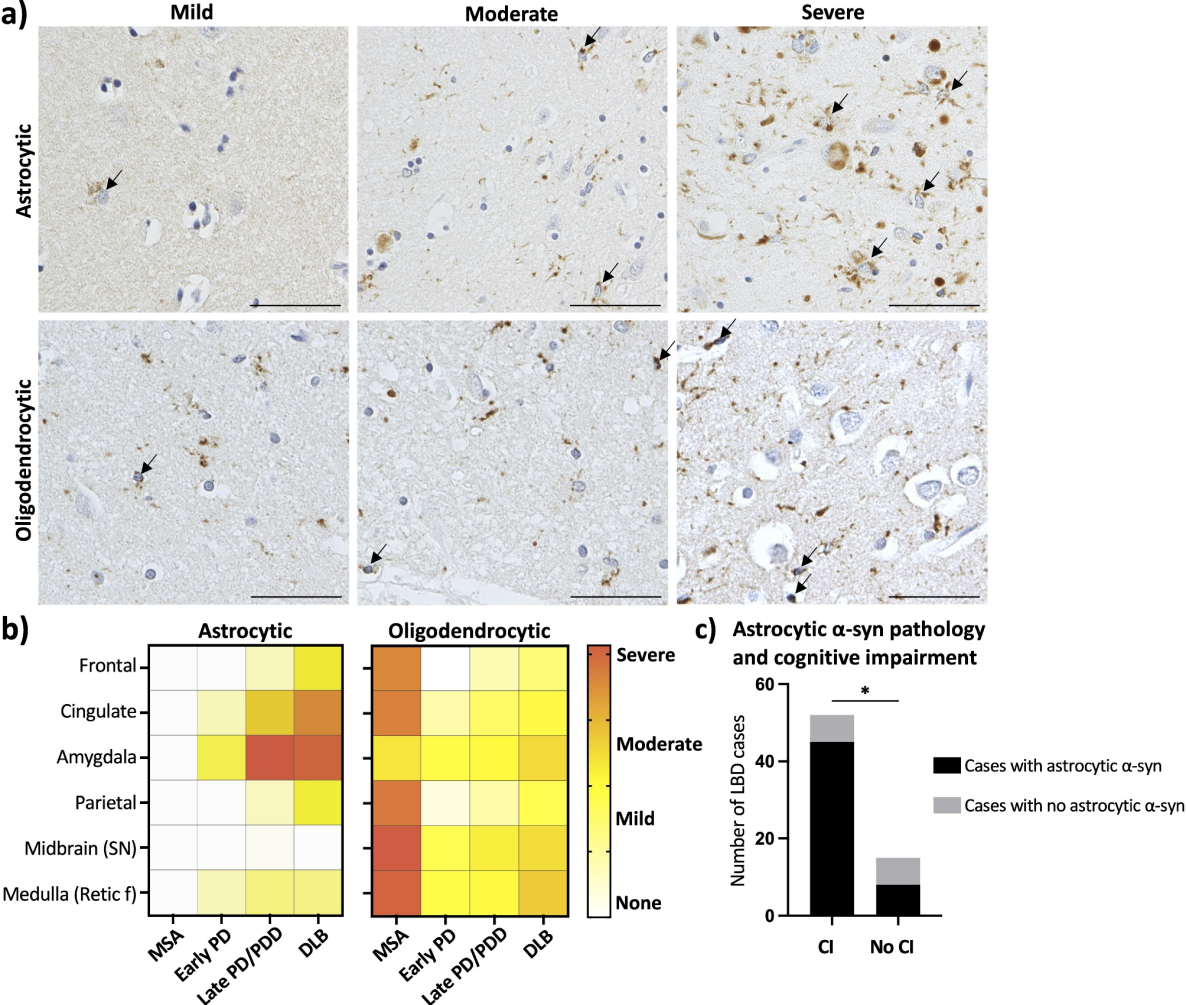
Astrocytic and oligodendrocytic α-syn pathology severity and prevalence were classified using a semi-quantitative scoring template. The severity of astrocytic and oligodendrocytic α-syn pathology was categorized into absent, mild (1 inclusions per 40x field of view), moderate (2–3 inclusions per 40x field of view) or severe (more than 3 inclusions per 40x field of view), according to the density of labeling. All regions across cases (MSA, early PD, late PD/PDD and DLB) were classified accordingly. Scale bar is 100 μm (**a**). A summary heat-map representing the severity of astrocytic and oligodendrocytic α-syn pathology across brain region and condition is shown (**b**). Astrocytic α-syn pathology was more abundant in LBD cases with cognitive impairment (CI), Fisher’s exact test (*p* < 0.05) (**c**)

The post-mortem neuropathological analysis revealed substantial glial α-syn pathology which appeared in a region- and disease-specific manner. To quantify cases with glial pathology, we developed a semi-quantitative scale to designate oligodendrocytic and astrocytic α-syn inclusions as mild (1 inclusions per 40x field of view), moderate (2–3 inclusions per 40x field of view) or severe (more than 3 inclusions per 40x field of view) (Fig. 1a). Oligodendrocytic α-syn inclusions were found in regions affected by α-syn at each stage. The pattern observed in astrocytic α-syn pathology was distinct and these inclusions were investigated in more detail. Negligible astrocytic α-syn pathology was found in any of the MSA cases across the analyzed regions. As a result, MSA cases were omitted from the subsequent sections which focus specifically on astrocytic α-syn pathology (Fig. 1b).

The amygdala was heavily involved in LBD cases, with astrocytic α-syn pathology appearing as early as in PD Braak stage 3 and progressively increasing in severity alongside advancing PD Braak stages. The cingulate cortex was the second most affected region, showing more severe involvement in DLB cases and with gradual progression from early PD to late PD/PDD cases. The frontal and parietal cortices were affected in cases exhibiting the most widespread and severe astrocytic α-syn pathology in the regions previously described. Interestingly, astrocytic α-syn pathology was rare in the brainstem, seldom observed in the SN (at levels below the threshold for mild according to our semiquantitative scale) and only mildly present in the reticular formation of the medulla though it was present throughout all PD Braak stages (Fig. 1b).

Since astrocytic α-syn pathology was restricted to a subset of cases and specific brain regions, we investigated the relationship between astrocytic α-syn pathology and clinical parameters, such as age of onset, disease duration and presence of cognitive impairment in LBD. Our analysis revealed that significantly, more cognitively impaired cases had mild astrocytic α-syn pathology in at least two regions, or moderate astrocytic α-syn pathology in one region. Interestingly, no correlation was found with disease duration or age of onset (Fig. 1c).

### Novel morphological classification of astrocytic α-syn pathology correlates with the severity of involvement

**Fig. 2:**
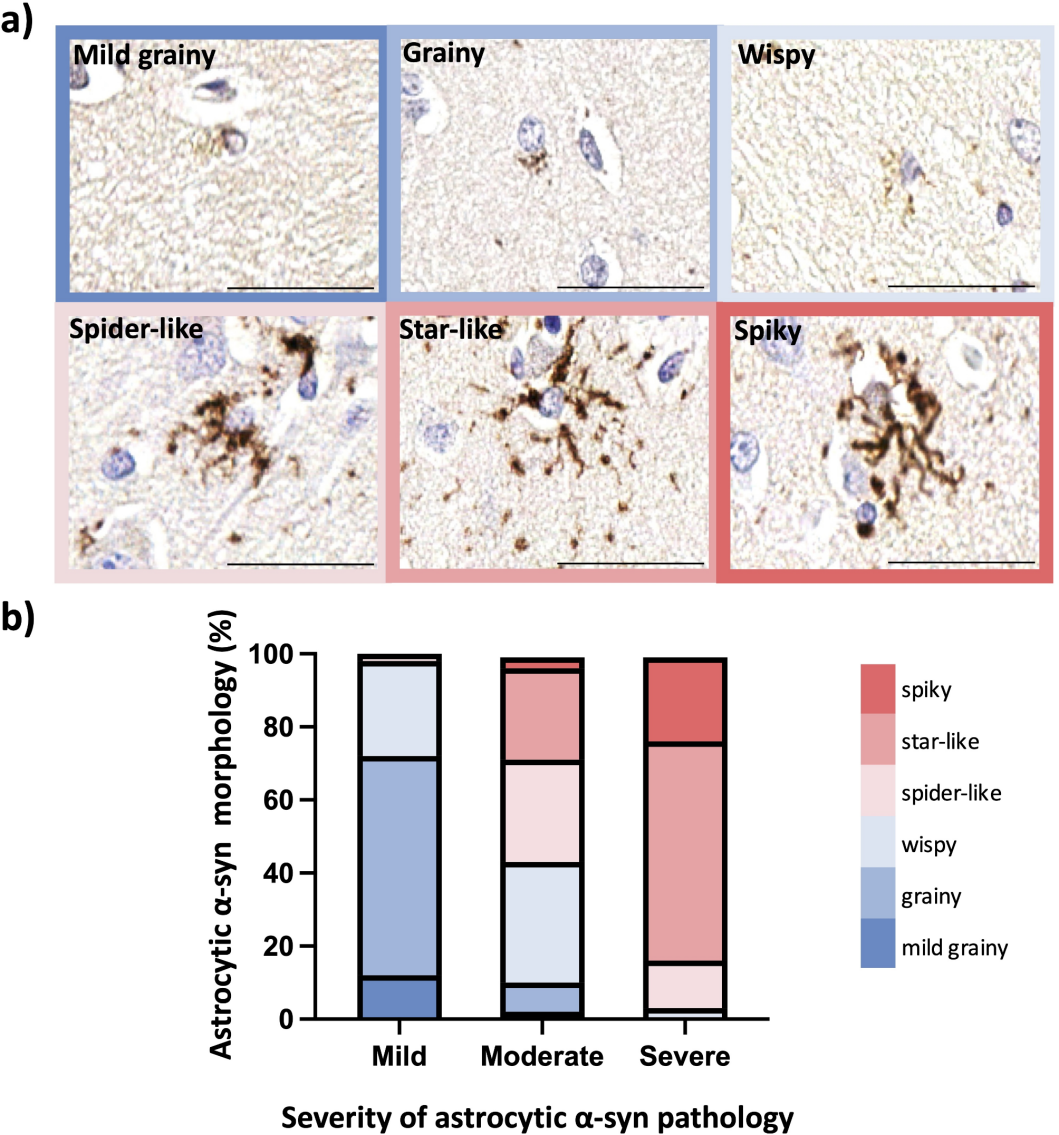
Classification and correlation of novel morphologies of astrocytic α-syn inclusions with severity in LBD. Astrocytic α-syn inclusions were classified into mild grainy, grainy, wispy, spider-like, star-like and spiky morphologies. Scale bar is 50 μm (**a**). The percentage of each type of astrocytic inclusion morphology across the three severity stages of astrocytic α-syn pathology is shown in stacked bars (**b**). The graph is a composite of PD/PDD and DLB cases where all the brain regions of each case were classified individually according to the severity of astrocytic α-syn pathology, and the predominant morphology was used for the analysis

Upon further investigation of the astrocytic α-syn inclusions in LBD, we identified a number of different morphologies. In brief, ‘grainy’ inclusions were those contiguous to the nucleus, presenting as condensed granular-like structures restricted to a single point in the cytoplasm. ‘Mild grainy’ were morphologically similar to grainy but smaller and less defined. ‘Wispy’ astrocytic α-syn inclusions displayed granular-like, thin and short ramifications elongating further than the grainy inclusions and not restricted to protruding from a single cytoplasmic point. ‘Spider-like’ had longer, thicker ramifications covering no more than half of the cytoplasm, with small hair-like projections emanating from the main ramifications. ‘Star-like’ were centrifugal, radiating from the nucleus as straight projections while ‘spiky’ astrocytic α-syn inclusions formed a mesh-like morphology with rigid, thick ramifications sometimes with an indistinct nucleus (Fig. 2a). These morphologies were confirmed to be found in astrocytes via double labeling with GFAP (see Supplementary Fig. 3, Additional file 1). Oligodendrocytic α-syn inclusions did not display a clear pattern in terms of morphology, severity or progression and thus were not further explored.

The morphology of astrocytic α-syn inclusions correlated with the severity of astrocytic α-syn pathology. Specifically, regions and cases with mild involvement predominantly displayed mild grainy and grainy morphologies. In contrast, moderately affected cases mainly exhibited wispy and spider-like astrocytic α-syn inclusions. In most affected areas and cases, morphologies such as star-like and spiky commonly appeared alongside grainy- and wispy-like forms suggesting a concomitant progression in the morphology and severity of astrocytic α-syn pathology (Fig. 2b).

**Fig. 3:**
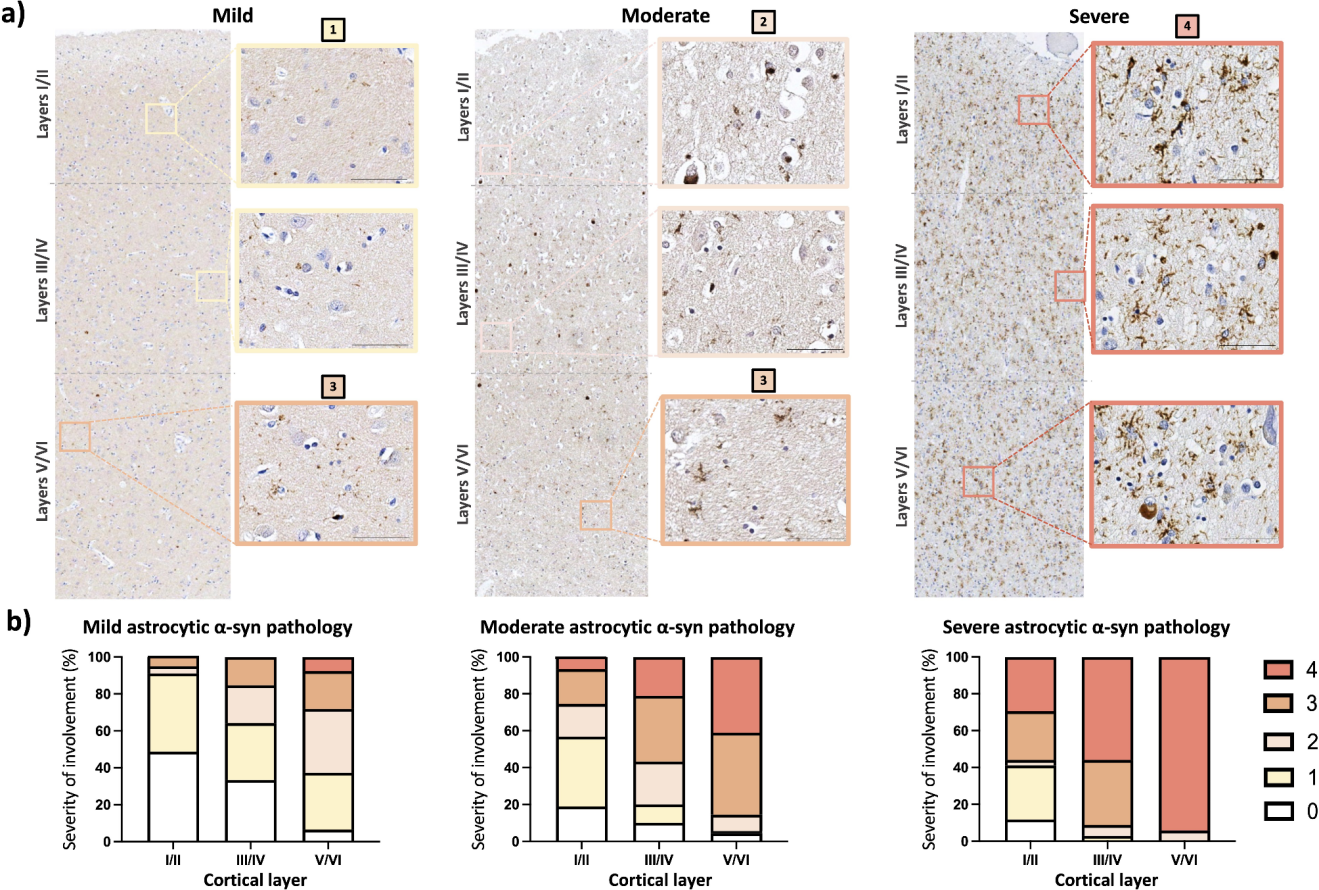
Cortical layers V/VI were affected first, and more heavily, by astrocytic α-syn pathology. Cases were classified, according to severity based on labeling density, into mild, moderate or severe astrocytic α-syn pathology as previously described, and the severity of involvement was analyzed per cortical layer and ranged from 0–4 (0 = absent, 1 = minimal pathology, 2 = scattered astrocytic α-syn pathology with grainy morphology, 3 = modest astrocytic α-syn pathology and wispy-like and 4 = any with high levels of astrocytic α-syn pathology in spider-, star-like and spiky morphologies). Representative pictures of cortical layers (subdivided into layers I/II, III/IV and V/VI) classified from 0–4 are shown as insets, across the severities of astrocytic α-syn pathology. Scale bar is 100 μm (**a**). The percentage of cases with each level of severity across cortical layers, ranging from 0–4 is shown for each overall severity of astrocytic α-syn pathology (mild, moderate, severe) irrespective of the condition (PD/PDD or DLB) and cortical region (**b**). *Cing* (cingulate), *Fr* (frontal cortex), *Par* (parietal), *ECx* (entorhinal cortex)

The relationship between the morphology and the severity of astrocytic α-syn inclusions was retained across the cortical layers (frontal, parietal, cingulate or entorhinal cortices). In early stages of astrocytic α-syn pathology, cortical layers V/VI were affected first with mild grainy and grainy morphologies, although occasionally wispy and spider/star-like morphologies were observed. In moderately affected cases, cortical layer V/VI continued to predominantly present wispy and spider/star-like morphologies, and increasingly involved layers III/IV. In cases with severe astrocytic α-syn pathology, all cortical layers were severely affected most prominently in layers V/VI, followed by intermediate layers III/IV and lastly by the most superficial layers I/II (Fig. 3), (see Supplementary Fig. 4, Additional file 1).

### The neuroanatomical distribution and the stepwise progression of astrocytic α-syn pathology differs from that in neurons and oligodendrocytes

**Fig. 4:**
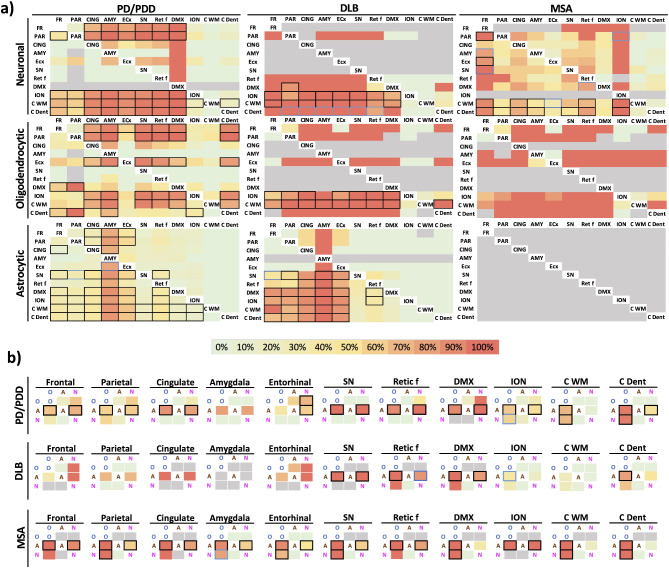
Conditional probability matrices for the regional and cell-type specific α-syn pathology. Summarized conditional probability analyses indicating the likelihood of astrocytic, neuronal, oligodendrocytic pathology in a brain region preceding another region in PD/PDD, DLB and MSA are shown. Results show the probability that the region above has α-syn pathology in absence of α-syn pathology in the region on the left. The probability percentage is shown as a color scale with red indicating a probability of 100% (**a**). The conditional probability matrices display the likelihood of a cell type containing α-syn inclusions before another (**b**). The color scale of the likelihood-percentage is shown, and percentages with black squares represent *p* < 0.05 and blue squares represent *p* < 0.09. Values in grey represent those with non-calculable differences. *Fr* (frontal), *Par* (parietal), *Cing* (cingulate), *Amy* (amygdala), *ECx* (entorhinal), *SN* (substantia nigra), *Retic f* (reticular formation, medulla), *DMX* (dorsal motor nucleus X), *ION* (inferior olivary nucleus), *C WM* (cerebellar white matter), *C Dent* (cerebellar dentate)

After unveiling extensive glial α-syn pathology in LBDs, our aim was to investigate the stepwise neuroanatomical distribution and the temporal involvement of each cell-type specific α-syn inclusion. To achieve this, a conditional probability matrix was implemented to evaluate the probability that one brain region exhibits α-syn pathology (in each cell separately) given that another brain region does not. In essence, the probability of a brain region preceding another, as previously described [21, 24]. Oligodendrocytic, astrocytic and neuronal α-syn pathologies were analyzed separately in PD/PDD (including both early PD and late PD/PDD), DLB and MSA cases. Overall, the results demonstrated that in MSA cases, neuronal α-syn pathology in the frontal cortex and ION preceded most other regions such as the cerebellum and the parietal cortex. Since astrocytic α-syn inclusions were insignificant in MSA, the conditional probability could not be calculated. In DLB cases, the general pattern revealed that neuronal and oligodendrocytic α-syn pathology followed similar progression trajectories, with regions such as the amygdala, cingulate cortex, SN and medullary reticular formation most likely preceding the ION and the cerebellum. Similarly, oligodendrocytic and neuronal α-syn pathology in PD/PDD cases exhibited a parallel distribution with regions such as the DMX, medullary reticular formation, SN and amygdala preceding most brain regions and were followed by the cingulate and neocortex. In contrast, astrocytic α-syn pathology in LBD featured a distinct progression with DLB cases exhibiting a strong focus in the limbic system (amygdala, cingulate and entorhinal cortices), whereas, PD/PDD cases displayed an amygdala-centric pattern. In PD/PDD cases, astrocytic α-syn pathology in the amygdala was followed by the cingulate and entorhinal cortices. These regions were highly likely to precede appearance of astrocytic α-syn pathology in the medullary reticular formation and the neocortex in DLB and PD/PDD cases (Fig. 4a).

A conditional probability matrix was implemented to evaluate the likelihood of one cell-type specific α-syn pathology preceding another. In PD/PDD, neuronal and oligodendrocytic α-syn pathology were highly likely to precede the appearance of astrocytic α-syn inclusions in most brain regions. Nevertheless, in relatively spared regions such as the ION and cerebellum, oligodendrocytic α-syn pathology was observed to occur primarily. A comparable pattern was seen in DLB cases. Unsurprisingly, oligodendrocytic α-syn pathology in MSA consistently preceded neuronal α-syn pathology (Fig. 4b).

**Fig. 5:**
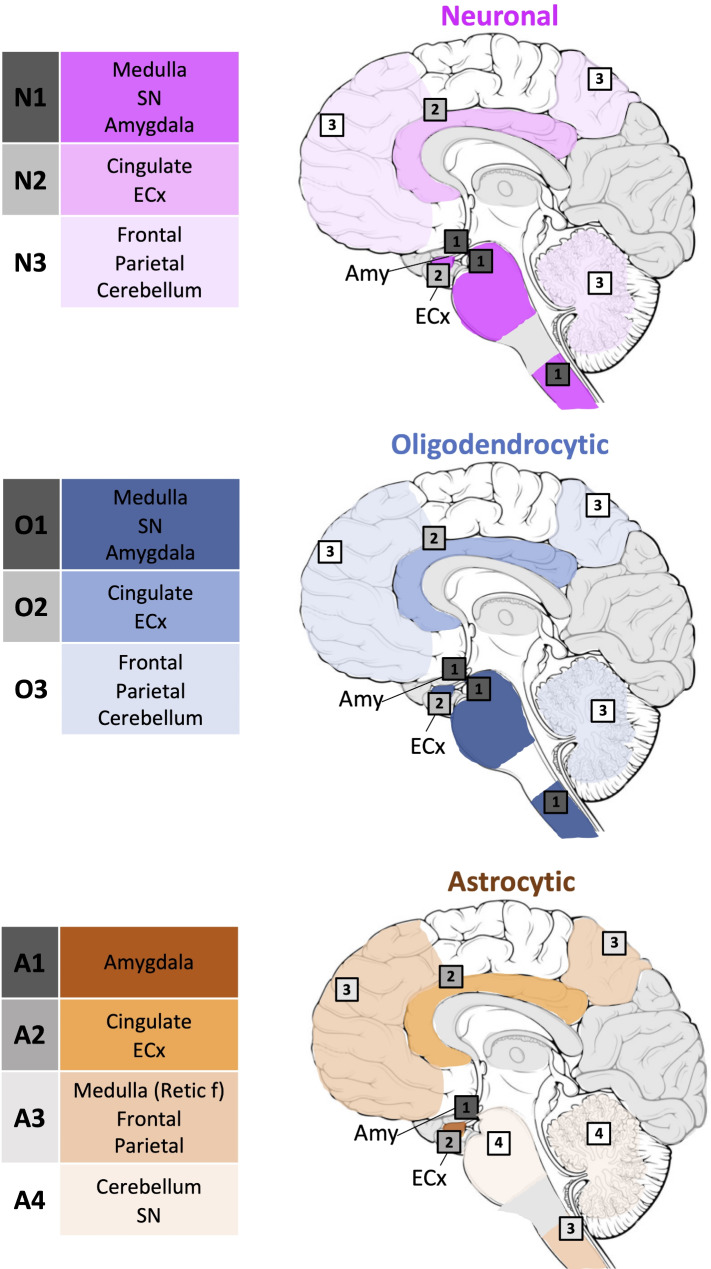
Proposed combined model considering the stepwise neuroanatomical distribution of cell-type specific α-syn pathology in PD/PDD. Neuronal and oligodendrocytic α-syn pathology first appeared in the medulla (DMX and reticular formation), SN and amygdala, followed by the cingulate and entorhinal cortex. The neocortex (frontal and parietal cortices) and the cerebellum were affected last. The hierarchical pattern of involvement in astrocytic α-syn pathology was unique. The amygdala is affected first, followed by the cingulate and entorhinal cortices. Then, the neocortex (frontal and parietal cortices) and reticular formation in the medulla were affected. Lastly, the cerebellum and the SN were involved. *ECx* (entorhinal cortex), *SN* (substantia nigra)

Using data derived from the conditional probability matrices, we proposed an initial combined staging model for PD/PDD cases reflecting the differential stereotypical progression pattern of neuronal, oligodendrocytic and astrocytic α-syn pathology (Fig. 5). The sequential anatomical distribution of neuronal α-syn pathology were analogous to that of oligodendrocytic α-syn pathology and aligned closely with the established Braak staging system. Neuronal and oligodendrocytic α-syn pathology initially appeared in the medulla (DMX and reticular formation), SN and amygdala in stage 1 (N1 and O1). Subsequently pathology progressed to the cingulate and entorhinal cortices (N2 and O2) followed by the frontal, parietal cortices and the cerebellum (N3 and O3). The divergent distribution pattern of astrocytic α-syn pathology reflected the distinct nature of these inclusions. Astrocytic α-syn pathology first emerged in the amygdala (A1) followed by limbic structures such as the cingulate and entorhinal cortices (A2). Later stages involved the medullary reticular formation as well as the frontal, parietal cortices s (A3) before eventually reaching the cerebellum and SN (A4). Across cortical regions, all cell-type specific α-syn pathology adhered to a consistent hierarchical involvement of the cortical layers with deeper layers (layers V, VI) affected prior to the intermediate and superficial cortical layers.

### Machine learning algorithm reveals cell-type and region specific α-syn pathology profile

**Fig. 6:**
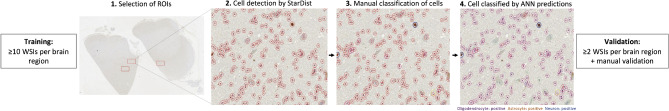
Machine learning algorithm accurately classified cell-type specific perikaryal α-syn inclusions across conditions and brain regions. The algorithm was trained with regions of interest (ROIs) selected in minimum 10 whole slide images (WSIs) per brain region including the midbrain (SN), medulla (reticular formation) amygdala and cortices. Then, StarDist was used as a deep-learning method for cell detection. The machine learning-based classifiers were developed using artificial neural networks (ANN) for the classification of cells into oligodendrocyte, astrocyte, neuron, oligodendrocyte: positive, astrocyte: positive or neuron: positive. Cells were manually labeled and classified, and unlabeled cells were classified by the real-time predictions made by the ANNs. The cell classification was validated by applying the algorithm into initially 2 WSIs (per brain region) and all WSIs were manually validated prior to analysis

To achieve a more granular and detailed understanding of the regional cell type-specific α-syn pathology across α-synucleinopathies, we applied machine learning methods to quantify perikaryal α-syn inclusions in oligodendrocytes, neurons, and astrocytes. Specifically, a machine learning algorithm was developed for the precise, accurate classification and automated detection of cell-type specific perikaryal α-syn inclusions. This workflow utilized QuPath [6] and StarDist software [49] leveraging artificial neural networks (ANN)-based classifiers to quantify α-syn inclusions across distinct cell types (Fig. 6). The categorization of α-syn inclusions in each cell type was conducted by microscopic examination of stained tissue sections guided by previously established descriptions alongside a visual template for reference (see Supplementary Fig. 1, Additional file 1). The classifiers were designed to enable differential detection of cell-type specific α-syn perikaryal inclusions across representative brain regions such as the brainstem (medulla (reticular formation) and midbrain (SN)), limbic system (amygdala and cingulate cortex) and neocortex (frontal and parietal cortices).

**Fig. 7:**
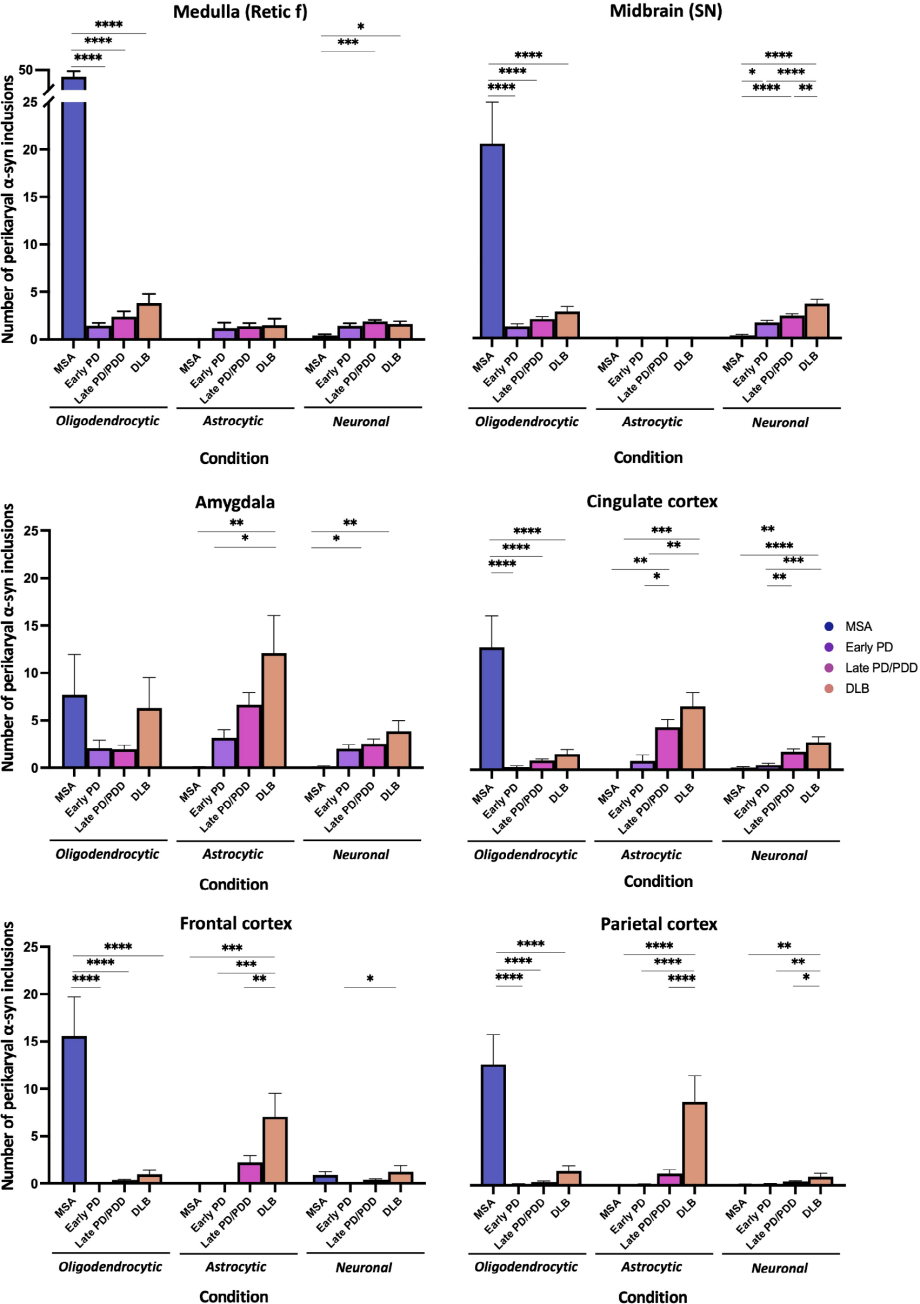
Machine learning algorithm quantification of cell-type specific α-syn inclusions across α-synucleinopathies. The number of oligodendrocytic, astrocytic and neuronal perikaryal α-syn inclusions detected using ANN-classifiers were compared between conditions in the brainstem (medulla – reticular formation, midbrain – SN), limbic system (amygdala and cingulate cortex) and neocortex (frontal and parietal cortices). The number of perikaryal α-syn inclusions per cell type were analyzed by one-way ANOVA followed by Tukey’s post-hoc test (*p < 0.05; ** *p* < 0.01; *** *p* < 0.001; **** *p* < 0.0001). Bars represent mean ± SEM and graphs have the same axis

The machine learning algorithm identified widespread glial α-syn pathology (oligodendrocytes and astrocytes) in LBD cases, whereas astrocytic α-syn pathology was seldom seen in MSA cases. As expected, oligodendrocytic α-syn pathology was significantly higher in MSA cases, except in the amygdala. Neuronal perikaryal α-syn inclusions were lowest in the medulla (reticular formation) of MSA with highest levels observed in the midbrain (SN) of DLB cases. Astrocytic α-syn pathology in the SN was mostly absent whilst similar levels of astrocytic α-syn were present amongst LBD cases in the medullary reticular formation. In the amygdala, astrocytic α-syn pathology was most prominent in DLB cases while neuronal α-syn inclusions were lowest in MSA. In the cingulate cortex, neuronal perikaryal α-syn inclusions were decreased in early PD as compared to late PD/PDD and DLB cases whilst astrocytic α-syn pathology was significantly lowered in early PD cases. α-Syn pathology was extensive in the neocortex of DLB cases, and neuronal α-syn inclusions were highest in DLB cases, particularly in the parietal cortex (Fig. 7).

### SuStaIn modeling revealed alternative progression patterns of cell-type specific α-syn pathology

To investigate the detailed temporal progression and potential heterogeneity in the progression of cell-type specific α-syn pathology in PD/PDD, while accounting for the severity of involvement, we implemented SuStaIn, an unsupervised machine learning-based approach [52]. SuStaIn modeling was applied separately to neuronal, oligodendrocytic and astrocytic α-syn inclusions across selected regions analyzed by the machine learning algorithm to quantify cellular α-syn inclusions (medulla (reticular formation), midbrain (SN), amygdala, cingulate, frontal and parietal cortices). We only focused on PD/PDD cases due to the limited number of DLB and MSA cases as well as the lack of astrocytic α-syn pathology in MSA. We first ran SuStaIn assuming a single progression trajectory and re-ran allowing for the identification of multiple progression patterns.

**Fig. 8:**
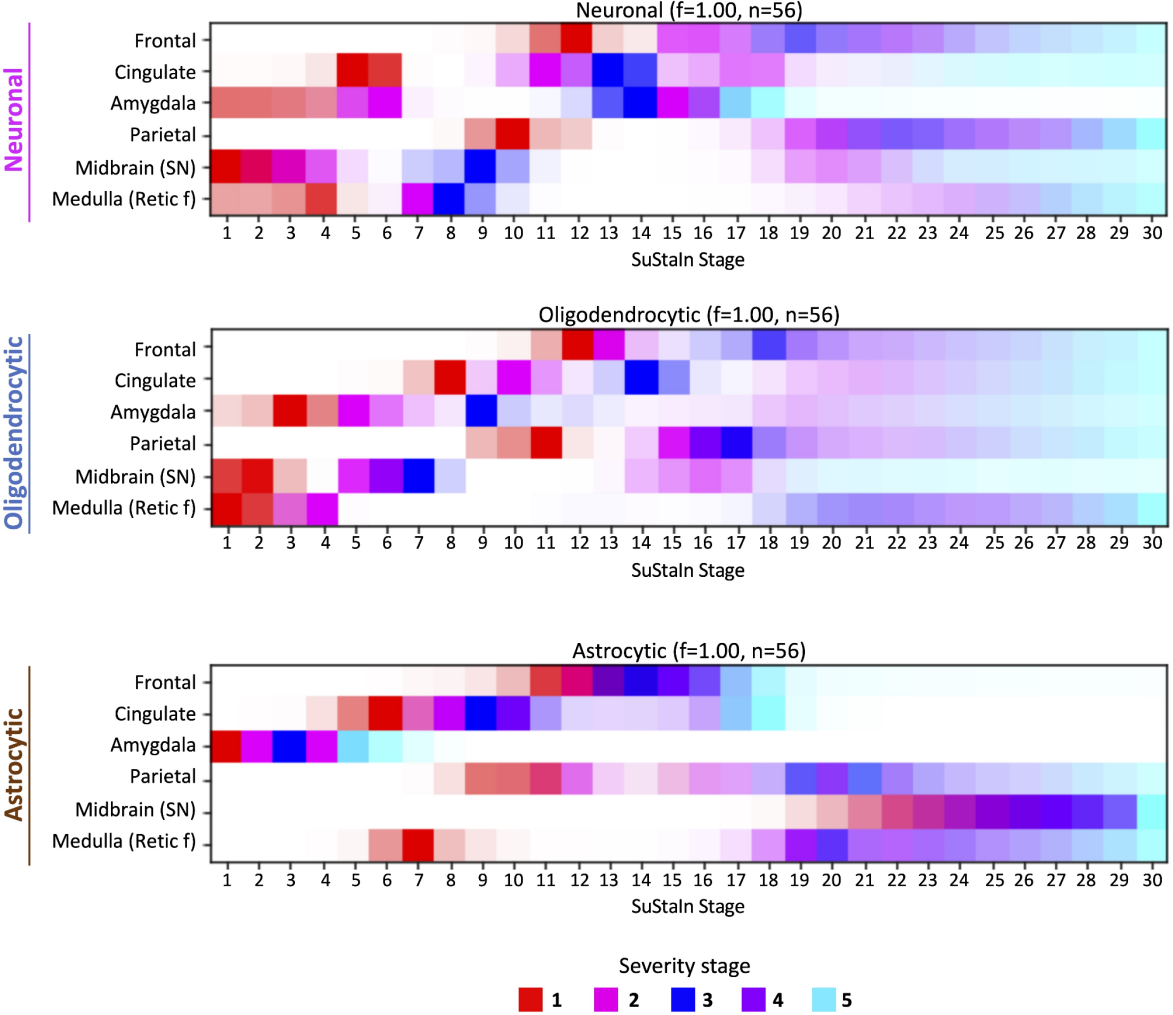
Neuroanatomical progression of cell-type specific α-syn pathology identified by SuStaIn modeling in PD/PDD cases. SuStaIn inferred the trajectory of neuronal, oligodendrocytic and astrocytic α-syn pathology progression in PD/PDD cases. SuStaIn stages are represented by color-scale depicting the sum of probabilities across severities (from 1–5). The intensity of the color depicts the certainty and confidence of the SuStaIn stage. *SN* (substantia nigra), *Retic f* (reticular formation, medulla)

Assuming one progression pattern: for neuronal α-syn, SuStaIn revealed a brainstem (midbrain (SN) and medullary reticular formation)-driven subtype which progressed to the amygdala and the cingulate cortex. Neocortical areas (parietal and frontal cortices) were affected last. A similar pattern of progression was detected with oligodendrocytic α-syn pathology, overall aligning with Braak staging [9] and our previous results (Fig. 4). Conversely, the astrocytic pattern of pathological spread was unlike the others. In detail, the amygdala was first affected, followed by the involvement of the cingulate cortex once the amygdala reached a severe stage (severity SuStaIn stage 4–5). The medullary reticular formation was then involved followed by the frontal and parietal cortices in the last stages (Fig. 8).

Assuming that not all cases might adhere to a single progression pattern, we re-applied the SuStaIn algorithm allowing for the identification of multiple progression patterns which only identified two subtypes. It revealed the presence of a major and minor subtype across all cell-type specific α-syn pathologies, with the major subtype reflecting the trajectory shown in (Fig. 8). For neuronal α-syn pathology, the majority of subjects followed the subtype identified from the single-progression pattern model. However, SuStaIn identified an alternative amygdala-driven subtype, which deviated with the early and heavier involvement of the amygdala alongside the SN, likely reflecting cases with amygdala-predominant α-syn pathology.

**Fig. 9:**
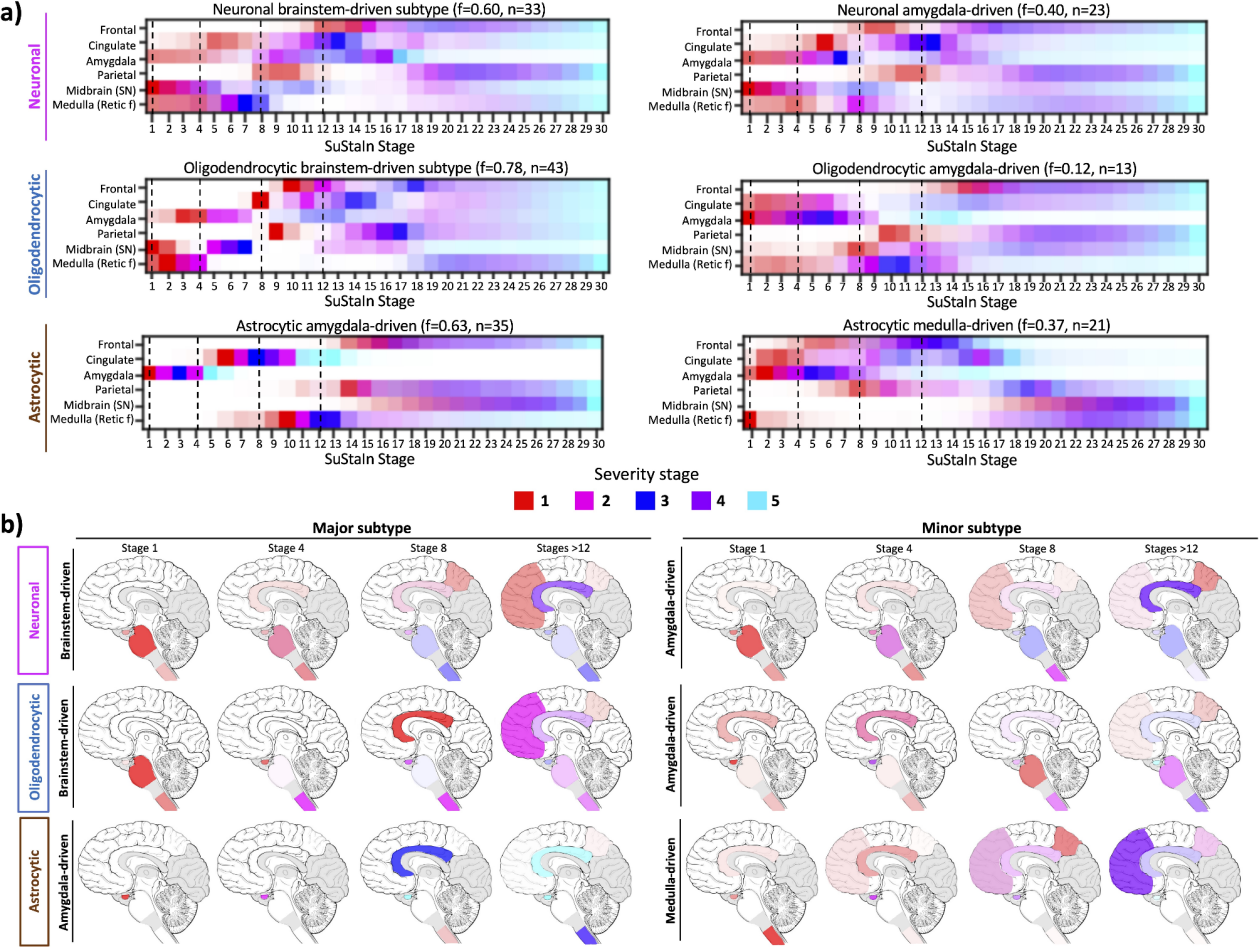
Heterogeneous progression patterns of cell-type specific α-syn pathology identified by SuStaIn analysis in PD/PDD cases. SuStaIn inferred two subtypes for the trajectory of neuronal, oligodendrocytic and astrocytic α-syn pathology. Neuronal and oligodendrocytic brainstem-driven and astrocytic amygdala-driven subtypes reflected the progression pattern in most cases. SuStaIn stages are represented by color-scale depicting the sum of probabilities across severity (from 1–5). The intensity of the color depicts the certainty and confidence of the SuStaIn stage (**a**). Representative diagrams of both subtypes pattern of progression in neuronal, oligodendrocytic and astrocytic α-syn pathology are illustrated. Stages 1, 4, 8 and > 12 are shown (**b**). *SN* (substantia nigra), *Retic f* (reticular formation, medulla)

The pattern of oligodendrocytic α-syn pathology in the brainstem-subtype was comparable to the brainstem-subtype of neuronal α-syn pathology, in agreement with our previous data. However, the amygdala-driven revealed an alternate progression pattern in which the oligodendrocytic α-syn pathology first appeared in the amygdala and cingulate cortex, progressing to the brainstem and neocortical areas (Fig. 9).

The pattern of astrocytic α-syn pathology deviated significantly from that observed in neuronal and oligodendrocytic α-syn pathology, congruent with the data from the conditional probability matrix. SuStaIn identified an amygdala-driven astrocytic α-syn subtype which accumulated to maximum severity before progressing the cingulate cortex, followed by the medullary reticular formation and ultimately to the parietal and frontal cortices. In contrast, the alternative subtype encompassed a minority of cases where astrocytic α-syn pathology was primarily observed in the medullary reticular formation before progressing to the amygdala, cingulate, frontal, parietal, and midbrain (SN). However, the pathology in the medullary reticular formation stagnated until later stages of the disease while the pathology in the amygdala rapidly and continuously progressed (Fig. 9).

### Newly proposed stages based on the stepwise neuroanatomical distribution of cell-type specific α-syn pathology

Here, we propose multimodal stages of PD/PDD considering the distinct progression pattern described for neuronal, oligodendrocytic and astrocytic α-syn pathology. By incorporating the inferred progression using SuStaIn, we have expanded on our previous model providing further detail and additionally subdividing the temporal events to create a refined model composed of 6 stages for each cell-type specific α-syn pathology. Most cases followed the progression pattern previously proposed in our model (Fig. 5) as shown by the conditional probability matrix (Fig. 4) which converged with SuStaIn modeling (Fig. 8). However, here we also describe the possibility that a minority of cases might follow an alternative pattern.

**Fig. 10:**
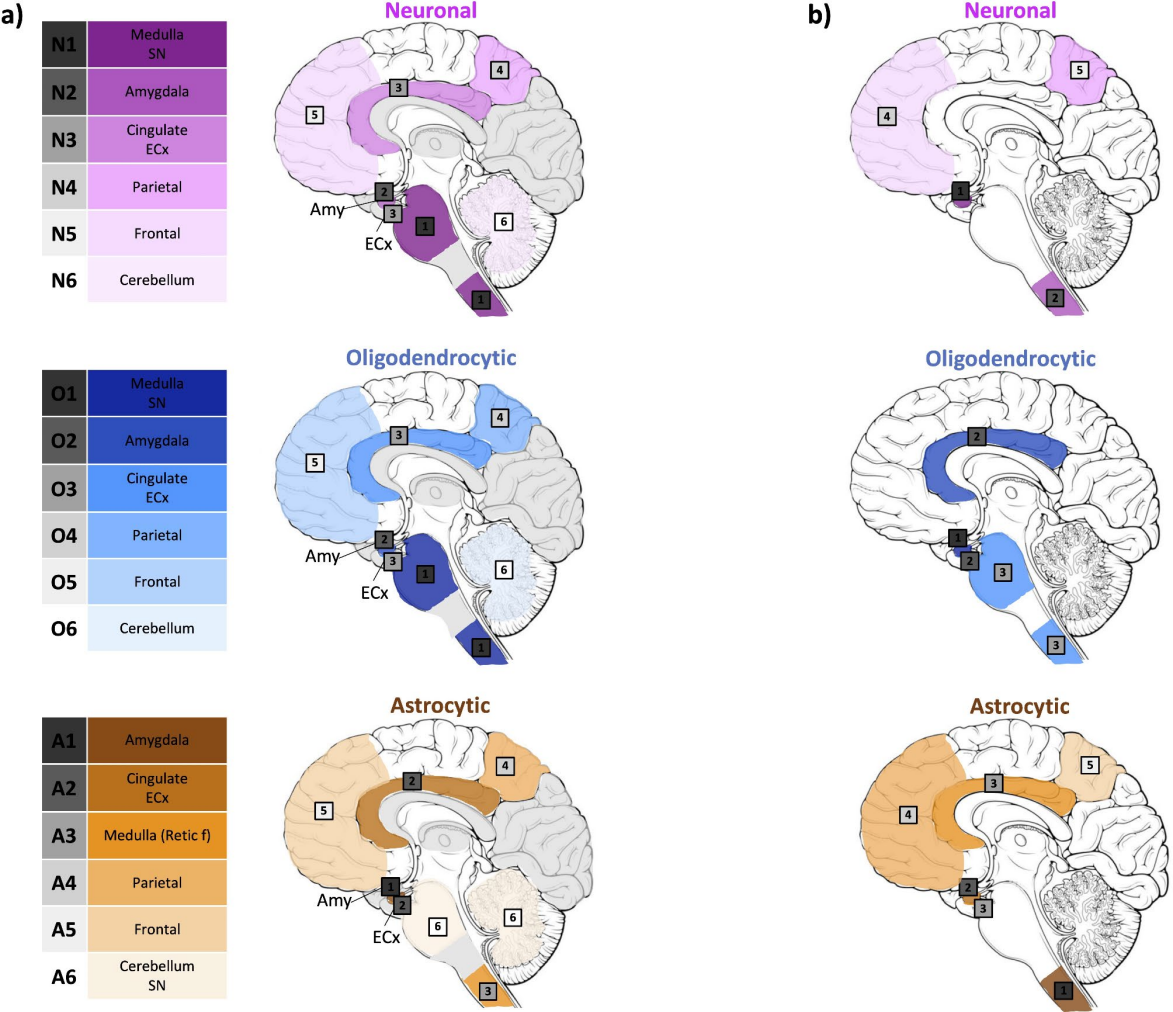
Refined combined model illustrating the stepwise neuroanatomical distribution of cell-type specific α-syn pathology in PD/PDD. Neuronal and oligodendrocytic α-syn pathology first appears in the medulla (DMX and reticular formation) and SN, progressing to the amygdala, followed by the cingulate and entorhinal cortices. The parietal cortex and then the frontal cortex is involved prior to the cerebellum. The progression of astrocytic α-syn pathology displayed a distinct pattern. The amygdala is primarily affected, followed by the cingulate and entorhinal cortices. In the following stages the reticular formation of the medulla, then the parietal cortex and the frontal cortex are affected. Ultimately, the cerebellum and the SN are involved (**a**). SuStaIn analysis revealed a minority of cases may follow an alternative spread pattern. Differentially, neuronal α-syn pathology might start in the amygdala prior to the involvement of the medulla (reticular formation) and SN. Oligodendrocytic α-syn pathology would also appear first in the amygdala before progressing to the cingulate and entorhinal cortices and subsequently reaching the reticular formation of the medulla and SN. The progression pattern of astrocytic α-syn pathology could alternatively first affect the reticular formation of the medulla and then the amygdala. Only regions affected at different stages from those shown in Fig. 10a are highlighted (**b**). *SN* (substantia nigra), *ECx* (entorhinal)

The stepwise progression of neuronal α-syn pathology starts in the brainstem, including the medulla and the SN (N1). Neuronal pathology propagates to the amygdala (N2) and extends to other limbic regions such as the cingulate and entorhinal cortices (N3). The involvement of the neocortex begins with the parietal cortex (N4), and the frontal cortex (N5) before reaching the cerebellum (N6). Once again, we demonstrate that the neuroanatomical hierarchical distribution of oligodendrocytic α-syn pathology was largely analogous to neuronal α-syn pathology. Contrastingly, the spatiotemporal progression of astrocytic α-syn pathology is unique. The amygdala is first affected (A1) followed by the cingulate and entorhinal cortices (A2). Then, the medullary reticular formation is affected (A3) prior to the progression of α-syn pathology to the parietal (A4) and frontal (A5) cortices. The cerebellum and SN are affected last (A6) (Fig. 10a).

In this model we also considered the alternative progression pattern of cell-type specific pathology displayed by the minor subtype revealed by SuStaIn modeling (Fig. 9b). In the alternative progression model, the amygdala exhibits extensive neuronal α-syn pathology at the same time as the SN, likely representing the pattern observed in amygdala-predominant cases. The remaining brain regions were affected in a manner consistent with the previously described sequential involvement, except for the frontal cortex being affected slightly prior to the parietal cortex. Conversely, the alternate model of oligodendrocytic α-syn pathology similarly unveils pathology commencing in the amygdala but subsequently involving the limbic system prior to the brainstem. The distinct progression pattern of astrocytic α-syn pathology describes pathology starting in the reticular formation of the medulla, followed by the amygdala and then the cingulate and entorhinal cortices. As revealed by the minor subtype, the frontal cortex appears to be affected slightly before the parietal cortex (Fig. 10b).

### Unified novel multimodal PD/PDD staging model of spatial, temporal and cellular involvement of α-syn pathology

**Fig. 11:**
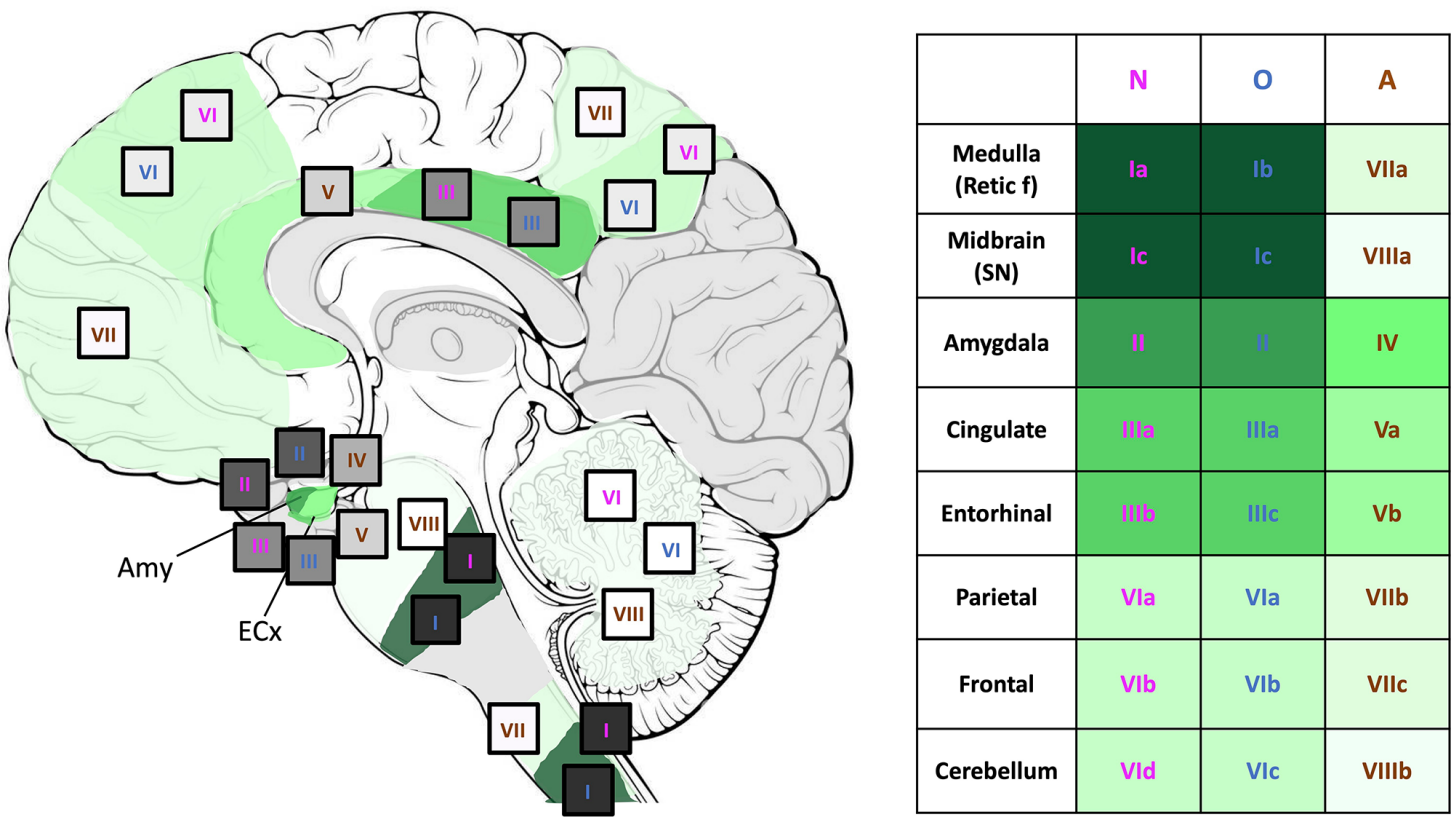
Novel multimodal stages describing the neuroanatomical distribution of cell-type specific α-syn pathology in PD/PDD cases. The model considers the data from the conditional probability matrix and SuStaIn analysis for the description of the stages based on the temporal progression of each cell-type α-syn pathology. In stage I, neuronal α-syn pathology first appears in the medulla (Ia), followed by oligodendrocytic pathology (Ib) and the presence of neuronal and oligodendrocytic α-syn pathology in the SN (Ic). In stage II, the amygdala is affected by neuronal and oligodendrocytic α-syn pathology. In stage III, neuronal and oligodendrocytic α-syn progresses to the cingulate cortex (IIIa), followed by the presence of neuronal (IIIb) and oligodendrocytic (IIIc) pathology in the entorhinal cortex. In stage IV, astrocytic α-syn pathology first emerges in the amygdala. In stage V, astrocytic α-syn pathology reaches the cingulate (Va) and entorhinal cortices (Vb). In stage VI, the neocortex is affected by neuronal and oligodendrocytic α-syn pathology first appearing in the parietal (VIa) and frontal (VIb) cortices. In stages VII, astrocytic α-syn pathology is progressively found in regions such as the reticular formation of the medulla (VIIa), parietal (VIIb) and frontal cortices (VIIc). Lastly, astrocytic α-syn pathology might appear in the SN (VIIIa) and in the cerebellum (VIIIb). *Retic f* (reticular formation, medulla), *SN* (substantia nigra), *N* (neuronal), *O* (oligodendrocytic), *A* (astrocytic)

To contextualize the neuroanatomical distribution of cell-type specific α-syn pathology in PD/PDD, we proposed a unified model that integrates both the spatial and temporal progression patterns implementing data from the conditional probability matrix (both regional and cellular; (Fig. 4a, b) and SuStaIn (Fig. 8)). The model incorporates the major subtype progression pattern for broad application within the research community, although it cannot be excluded that a minority might follow an alternative trajectory. The unified model incorporates altogether the stepwise neuroanatomical distribution of the uncovered oligodendrocytic and astrocytic alongside neuronal α-syn pathology as described in the combined staging model of the dominant subtype (Fig. 10) culminating in 8 main stages (Fig. 11). Our results display the initial involvement of neurons, closely followed by oligodendrocytes. Astrocytic α-syn pathology accumulates only once a certain threshold of neuronal and oligodendrocytic α-syn pathology is reached in the limbic areas such as the amygdala, cingulate and entorhinal cortices.

The unified model consists of 8 main stages which were further subclassified (a-c) as necessary. As a note, the presence of α-syn pathology in the cell-type or region stated in any subclassification (a-d) is sufficient to attribute a case into the corresponding main stage. Overall, stages I-III align with α-syn pathology described in neurons across PD Braak stages 1–4 but in our model involves both neurons and oligodendrocytes initially in the brainstem and subsequently progressing to the limbic system. In stages IV-V, α-syn pathology first accumulates in astrocytes in the amygdala and extends out to other limbic regions. Finally, stages VI-VII are defined by the involvement of the neocortex and other sporadically affected brain regions.

Stage I of the sequence begins with the involvement of the brainstem, where pathology commences in the medulla, affecting neurons (Ia), and oligodendrocytes (Ib) as well as in the SN (Ic). In stage II, neuronal and oligodendrocytic α-syn pathology extends to the amygdala. Stage III marks the progression of neuronal and oligodendrocytic α-syn to other limbic areas such as the cingulate cortex (IIIa), followed by the neuronal (IIIb) and oligodendrocytic (IIIc) pathology in the entorhinal cortex.

Stages I-III are uniquely characterized by the emergence of neuronal and oligodendrocytic α-syn pathology in the brainstem and limbic areas. Stage IV marks the first appearance of astrocytic α-syn pathology in the amygdala. By stage V, astrocytic α-syn is also observed in the cingulate (Va) and entorhinal cortices (Vb).

Stage VI is defined by neocortical involvement corresponding to the last Braak stages. In this stage, neuronal and oligodendrocytic α-syn inclusions appear in the parietal (VIa) and frontal (VIb) cortices, as well as the cerebellum, firstly affecting oligodendrocytes (VIc) and then neurons (VId). Stage VII is characterized by further progression of astrocytic α-syn pathology to the reticular formation of the medulla (VIIa), as well as the parietal (VIIb) and frontal cortices (VIIc). Finally, stage VIII is defined by the rare occurrence of astrocytic α-syn pathology in the SN (VIIIa) and the cerebellum (VIIIb).

## Discussion

Traditionally, LBD staging has primarily focused on the neuronal component of pathology; however recent efforts have shifted towards α-syn accumulations in glia. We have identified extensive astrocytic and oligodendrocytic α-syn pathology in LBD whereas astrocytic α-syn inclusions were seldom seen in MSA. To further investigate, we explored the breadth and the spatiotemporal distribution of oligodendrocytic, astrocytic and neuronal α-syn pathology in PD/PDD using semi-quantitative neuropathological analysis enhanced with automated machine learning modeling. Leveraging the distinct characteristics of cell-type specific α-syn pathology, we introduce a novel multimodal neuropathological framework incorporating the neuroanatomical progression and temporal events of oligodendrocytic and astrocytic with neuronal α-syn pathology in PD/PDD.

We first resolved the extent and spatiotemporal progression of glia α-syn pathology which was introduced alongside neuronal α-syn pathology. The progression pattern of α-syn pathology in oligodendrocytes was found to be analogous to that of neurons, whilst astrocytic α-syn pathology emerged later and manifested a unique progression pattern. Our findings further revealed that: (1) the development of astrocytic α-syn inclusions predominantly requires the presence of neuronal and oligodendrocytic α-syn pathology in the limbic system, and (2) the amygdala serves as a hotspot for astrocytic α-syn pathology. We propose a novel staging model that not only offers a more detailed framework for understanding the progression but also elucidates key pathomechanistic underpinnings of α-syn pathology. The proposed staging model first describes the appearance of neuronal and oligodendrocytic α-syn pathology in the brainstem (I) and the limbic system (II-III). Astrocytic α-syn inclusions initially arise in the amygdala (IV) as well as in the cingulate and entorhinal cortices (V). Neuronal and oligodendrocytic α-syn pathology then progresses to neocortical areas and cerebellum (VI). Subsequently, astrocytic α-syn pathology spreads to neocortical regions and the medullary reticular formation (VII) and finally to the SN and the cerebellum (VIII).

We have unveiled widespread oligodendrocytic α-syn inclusions in LBDs exhibiting a stereotypical progression pattern parallel to that of neuronal α-syn pathology. Single-cell transcriptomic studies have reported an altered oligodendrocyte profile in PD [1, 5] with early changes occurring prior to neurodegeneration [11]. However, we showed that in some regions such as the ION and cerebellum, oligodendrocytic α-syn pathology may precede neuronal α-syn pathology, although it cannot be excluded that neurons harboring α-syn pathology might have already been lost. Literature has reported neuron-to-oligodendrocyte transfer which is supported by expression of α-syn by neurons [40]. Despite this, α-syn expression in oligodendrocytes remains controversial as it has mostly been observed in immature oligodendrocytes [16]. Although neurons are thought to be affected by α-syn pathology prior to oligodendrocytes our data raise the possibility that neurons and oligodendrocytes are affected simultaneously, both actively participating in disease pathogenesis and subsequent neurodegeneration. This complexity underscores the need to clarify the functional relationship between neuronal and glial α-syn interplay across α-synucleinopathies as well as how this dual involvement could potentially influence the clinical manifestations.

Our findings also uncovered the striking neuroanatomical distribution of astrocytic α-syn inclusions which succeeded neuronal and oligodendrocytic α-syn pathology. The stepwise progression of astrocytic α-syn pathology in LBD involved the amygdala, which was first and most severely affected. SuStaIn analysis revealed that moderate severity of astrocytic α-syn pathology (corresponding to SuStaIn severity stages 4–5) in the amygdala is required for propagation to other areas. Once established, astrocytic α-syn pathology rapidly progresses to the limbic system prior to neocortical α-syn pathology. Our results highlight the unique susceptibility of the amygdala, which is a focus for protein misfolding, including TDP-43, tau and beta-amyloid [35, 43] and it is extensively affected by α-syn pathology across all cell types. The neural connectivity of the amygdala might explain, at least partially, its central involvement as it is highly connected to other vulnerable regions in PD as well as those involved in cognition [48]. The hierarchical progression of astrocytic α-syn pathology does not follow the traditional caudo-rostral spread [9] but rather resembles the distribution of neuronal pathology in amygdala-predominant cases barring the involvement of the SN [8]. Additionally, a subset of PD/PDD cases exhibited a sequential progression of neuronal α-syn pathology characteristic of amygdala-predominant cases, suggesting that the early involvement of the amygdala may be underappreciated in current staging models. Our results agree with the Braak staging system [9] and corroborate previous studies indicating that at least one third of cases with Lewy pathology follow this amygdala-predominant pattern [39]. Similarly, a recent paper applying SuStaIn modeling revealed subtypes reflecting the early involvement of the limbic or brainstem although the cohort was large (*n* > 700) and included both PD/PDD and DLB cases thus explaining why the number of cases with early limbic involvement was higher than in our results [31]. However, this study was limited to neuronal pathology and did not consider it separately to the glial component. Further exploration of the significance of the amygdala in the early stages of α-synucleinopathies could unravel its crucial role in disease initiation and progression.

According to our model, astrocytes are affected at stage IV succeeding the establishment of neuronal and oligodendrocytic α-syn pathology in the brainstem and limbic system. This questions whether astrocytes might actively promote disease pathogenesis or merely serve as bystanders in the progression of α-syn pathology. The novel morphologies of astrocytic inclusions, which correlate with severity of involvement, are indicative of a potential neuroprotective role of astrocytes. These morphological variations likely reflect different stages of astrocyte activation in response to pathology [17] or could also potentially be related to astrocytic α-syn being truncated [3, 43]. Different astrocyte activation stages might translate into α-syn accumulation in different cellular compartments, influencing the fate and degradation rate of α-syn. Thus, not only does the density of astrocytic α-syn pathology provide insight into the overall extent of total α-syn pathology but the morphology of these inclusions may also offer valuable pathological information and potential mechanistic implications. By evaluating the density or the morphology of astrocytic α-syn pathology, we can speculate that cases with severe astrocytic α-syn involvement and/or star-like/spiky morphologies likely display neocortical α-syn pathology. The minimal presence of astrocytic α-syn inclusions in the SN, despite being the region most susceptible to degeneration in PD, may imply that the regional vulnerability is linked to aberrant astrocytic function [28]. Considering areas with the most and least number of astrocytic α-syn inclusions, neuronal loss in the amygdala is minimal (20–30%) as compared to the SN (where loss exceeds 60–70%). This disparity supports the hypothesis that astrocytes may exert a protective role in ameliorating neuronal death [18, 26]. In the context of negligible astrocytic α-syn pathology in MSA, this may also help explain why MSA manifests with the most aggressive and fastest disease course amongst all the α-synucleinopathies [25, 29]. Alternatively, given the paucity of neuronal α-syn pathology in MSA, it could be hypothesized that oligodendrocyte-to-astrocyte α-syn transfer is less efficient than that from neurons to astrocytes which may account for the absence of astrocytic α-syn inclusions in MSA. The neuron-to-astrocyte transfer of α-syn aggregates, which are subsequently targeted for degradation by astrocytes insinuates a potential neuroprotective mechanism aimed at impeding the spread of pathological α-syn [27, 30]. Astrocytes and microglia degrade pathological aggregates more efficiently [41] compared to oligodendrocytes (and neurons) which are more vulnerable to α-syn accumulation [22]. This difference in degradation capacity explains, at least partly, the build-up of α-syn aggregates in neurons and oligodendrocytes prior to astrocytes. It is important to note that a small proportion of microglia contained α-syn inclusions, although not categorized separately here. The accumulation of pathological α-syn in microglia has been reported in vivo [13] but seldom in post-mortem human tissue [3]. In vitro, co-cultures of microglia and astrocytes demonstrated their interaction in degrading α-syn aggregates with microglia exhibiting higher efficiency [41] which may explain the relative scarcity of microglial α-syn inclusions. Future research is required to specifically investigate the levels of microglia α-syn inclusions, their neuroanatomical distribution, and their relationship to other cellular α-syn pathology.

This study is not without limitations. Firstly, we acknowledge that post-mortem tissue does not allow for establishing a temporal sequence of events with absolute certainty. Although we cannot categorically affirm which areas are affected first during life, we can infer the likely trajectory. The early progression differentially identified by the minor subtype in SuStaIn modeling warrants further consideration. Additionally, it is important to note that a limited number of PD/PDD Braak stage 1 and 2 were analyzed, which means the temporal succession at the earliest stages might not be fully elucidated. Despite a range of early and late PD Braak stage cases, our cohort was derived exclusively from the Parkinson’s UK Brain Bank. Hence, the overall population might be biased towards advanced PD cases and limit the broader representation of the population. Additionally, we were unable to provide the same level of detail in MSA and DLB due to the lower prevalence of these cases and lack of early-stage samples. Finally, we cannot completely disentangle the individual contributions of α-syn accumulations in neurons, oligodendrocytes and astrocytes to clinical phenotype and pathological progression. In this study, we included representative brain regions encompassing the progression of α-syn from the brainstem to limbic regions and the neocortex, also including the cerebellum. Future work would require including other relevant brain regions such as the olfactory bulb, temporal cortex and the pons to fully comprehend the involvement of each cell type across the brain. Despite these limitations, the granularity of the regional and cell-specific involvement in PD/PDD cases facilitated the introduction of as well as the appreciation of glial pathology in a novel neuropathological staging model. Our data also challenges the previous staging systems and treatment approaches whose focus is solely on neuronal α-syn pathology. The role of glia in LBD pathogenesis described here warrants further exploration, as it may reveal potential therapeutic avenues to halt α-syn pathogenesis. Currently, the model described is intended for research purposes, introducing and resolving, for the first time, oligodendrocytes and astrocytes in the temporal succession of α-syn, although its applicability and clinical relevance should be further validated in the wider research community.

## Electronic supplementary material

Below is the link to the electronic supplementary material.

Supplementary Material 1

## Data Availability

No datasets were generated or analysed during the current study.
