# Supplementary material for: Neuropathological stages of neuronal, astrocytic and oligodendrocytic alpha-synuclein pathology in Parkinson’s disease
Source: Acta Neuropathol Commun. 2025 Feb 11;13:25. doi: 10.1186/s40478-025-01944-x (PMC11816504; doi:10.1186/s40478-025-01944-x)
Supplement: Supplementary file 1 — Supplementary Material 1 [file 40478_2025_1944_MOESM1_ESM.docx]

| **Disease** | **Gender** | **Age at death** | **Disease duration** | **α-syn** **(Braak)** | **Tau (Braak)** | **Beta-amyloid (Thal)** |
| --- | --- | --- | --- | --- | --- | --- |
| MSA | M | 86 | 8 | n.a. | I | 0 |
| MSA | M | 75 | 7 | n.a. | I | 0 |
| MSA | M | 53 | 7 | n.a. | 0 | 0 |
| MSA | M | 80 | 11 | n.a. | I | 0 |
| MSA | F | 85 | 9 | n.a. | V | 3 |
| MSA | F | 77 | 8 | n.a. | IV | 3 |
| MSA | F | 67 | 8 | n.a. | 0 | 3 |
| MSA | M | 66 | 4 | n.a. | II | 1 |
| MSA | F | 49 | 27 | n.a. | I | 0 |
| MSA | M | 71 | 14 | n.a. | III | 3 |
| MSA | F | 68 | 4 | n.a. | 0 | 2 |
| MSA | F | 91 | 5 | n.a. | 0 | 2 |
| MSA | F | 74 | 8 | n.a. | 0 | 0 |
| MSA | F | 70 | 5 | n.a. | II | 2 |
| MSA | F | 65 | 8 | n.a. | I | 0 |
| MSA | M | 69 | 3 | n.a. | 0 | 0 |
| MSA | M | 75 | 6 | n.a. | 2 | 0 |
| DLB | F | 81 | 14 | 6 | I | 2 |
| DLB | M | 61 | 14 | 6 | V | 2 |
| DLB | M | 58 | 15 | 6 | I | 2 |
| DLB | M | 70 | 1 | 5 | II | 2 |
| DLB | M | 63 | 9 | 6 | IV | 2 |
| DLB | F | 83 | 5 | 6 | IV | 2 |
| DLB | M | 76 | 6 | 6 | II | 2 |
| DLB | F | 82 | 5 | 6 | III | 3 |
| DLB | F | 76 | 5 | 6 | V | 3 |
| DLB | M | 77 | 3 | 6 | II | 3 |
| DLB | M | 70 | 11 | 6 | V | 3 |
| DLB | M | 89 | 5 | 6 | VI | 3 |
| DLB | M | 80 | 5 | 6 | VI | 3 |
| DLB | M | 86 | 10 | 6 | I | 0 |
| PD | F | 82 | 0 | 1 | II | 2 |
| PD | F | 98 | 0 | 2 | III | 3 |
| PD | F | 82 | 9 | 3 | I | 0 |
| PD | F | 93 | 14 | 3 | I | 0 |
| PD | F | 90 | 22 | 3 | II | 0 |
| PD | F | 66 | 8 | 3 | II | 1 |
| PD | M | 75 | 19 | 3 | II | 2 |
| PD | M | 87 | 9 | 3 | II | 1 |
| PD | F | 77 | 20 | 3 | n.a. | 0 |
| PD | M | 94 | 15 | 3 | II | 5 |
| PD | F | 76 | 10 | 4 | II | 0 |
| PD | F | 87 | 9 | 4 | I | 0 |
| PD | F | 75 | 9 | 4 | III | 5 |
| PD | M | 86 | 13 | 4 | III | 3 |
| PD | M | 77 | 1 | 4 | II | 3 |
| PD | M | 78 | 37 | 4 | II | 2 |
| PD | M | 78 | 9 | 4 | 0 | 0 |
| PD | M | 73 | 22 | 4 | I | 1 |
| PD | M | 71 | 7 | 5 | II | 0 |
| PD | M | 81 | 6 | 5 | II | 3 |
| PD | M | 79 | 15 | 5 | I | 1 |
| PD | F | 84 | 13 | 5 | III | 3 |
| PD | F | 84 | 16 | 5 | II | 1 |
| PD | M | 75 | 12 | 5 | II | 2 |
| PD | M | 80 | 20 | 5 | II | 2 |
| PD | F | 82 | 6 | 5 | II | 0 |
| PD | M | 88 | 10 | 5 | I | 0 |
| PD | M | 87 | 20 | 5 | III | 3 |
| PD | M | 82 | 7 | 6 | I | 2 |
| PD | M | 71 | 8 | 6 | II | 2 |
| PD | M | 84 | 6 | 6 | II | 3 |
| PD | F | 83 | 15 | 6 | IV | 5 |
| PD | M | 73 | 22 | 6 | II | 0 |
| PD | F | 83 | 31 | 6 | III | 3 |
| PD | F | 88 | 11 | 6 | III | 1 |
| PD | M | 87 | 10 | 6 | I | 0 |
| PD | F | 82 | 21 | 6 | III | 4 |
| PDD | F | 85 | 18 | 4 | III | 2 |
| PDD | M | 76 | 27 | 6 | II | 1 |
| PDD | F | 84 | 11 | 6 | IV | 2 |
| PDD | F | 76 | 18 | 6 | II | 2 |
| PDD | F | 83 | 8 | 6 | IV | 2 |
| PDD | M | 74 | 10 | 6 | II | 2 |
| PDD | F | 86 | 18 | 6 | IV | 3 |
| PDD | M | 83 | 16 | 6 | II | 1 |
| PDD | F | 89 | 7 | 6 | II | 3 |
| PDD | M | 78 | 8 | 6 | I | 0 |
| PDD | M | 84 | 9 | 6 | II | 3 |
| PDD | M | 77 | 9 | 4 | II | 3 |
| PDD | M | 78 | 21 | 5 | II | 0 |
| PDD | F | 88 | 7 | 3 | I | 0 |
| PDD | M | 68 | 17 | 4 | 0 | 2 |
| PDD | M | 88 | 10 | 4 | IV | 0 |
| PDD | F | 76 | 16 | 4 | 0 | 0 |
| PDD | F | 82 | 13 | 6 | III | 5 |

**Supplementary Table 1:** Detailed cohort information. Disease, gender, age at death, disease duration, α-syn (Braak staging), tau (Braak staging) and beta-amyloid (Thal phases) are reported.

**Supplementary Figure 1:** Visual template used for the categorization of oligodendrocytic, astrocytic and neuronal perikaryal α-syn inclusions. Pictures depict examples of perikaryal α-syn inclusions in oligodendrocytes, astrocytes and neurons that were used as templates for the detection of cell-type specific α-syn inclusions and training of the ANNs. Scale bar is 20µm.

**Supplementary Figure 2:** Immunofluorescence confirmed the astrocytic nature of astrocytic α-syn inclusions. Several astrocytic markers including GFAP, Glutamine synthase (GS), Aldh1l1and S100-B were used and co-localization with α-syn confirmed. White squares depict areas of co-localization while insets represent magnified inclusions. Scale bar is 100µm.

**Supplementary Figure 3:** Immunofluorescence validated the astrocytic origin of the various morphologies observed in astrocytic α-synuclein inclusions. GFAP was used as an astrocytic marker to confirm the astrocytic nature of grainy, wispy, spider-like and spiky morphologies, (b). White squares depict areas of co-localization while insets represent magnified inclusions. Scale bar is 50µm.

**Supplementary Figure 4:** Table explaining the score given for the astrocytic α-syn pathology in each cortical layer. Cases were classified into mild, moderate or severe astrocytic α-syn pathology as previously described, and the severity of involvement was analyzed in each cortical layer and ranged from 0-4 irrespective of the condition (PD/PDD or DLB) and cortical region. Assigned scores represent: 0 = absent, 1 = mildly affected cases with chiefly mild grainy astrocytic α-syn pathology, 2 = mildly or moderately affected cases with predominantly grainy astrocytic α-syn pathology, 3 = moderate and severe astrocytic α-syn pathology with primarily wispy-like morphology and 4 = moderate to severe levels of astrocytic α-syn inclusions with spider-, star-like and spiky morphologies.
